# Supplementary figures and images for: Structure of the HOPS tethering complex, a lysosomal membrane fusion machinery
Source: eLife. 2022 Sep 13;11:e80901. doi: 10.7554/eLife.80901 (PMC9592082; doi:10.7554/eLife.80901)

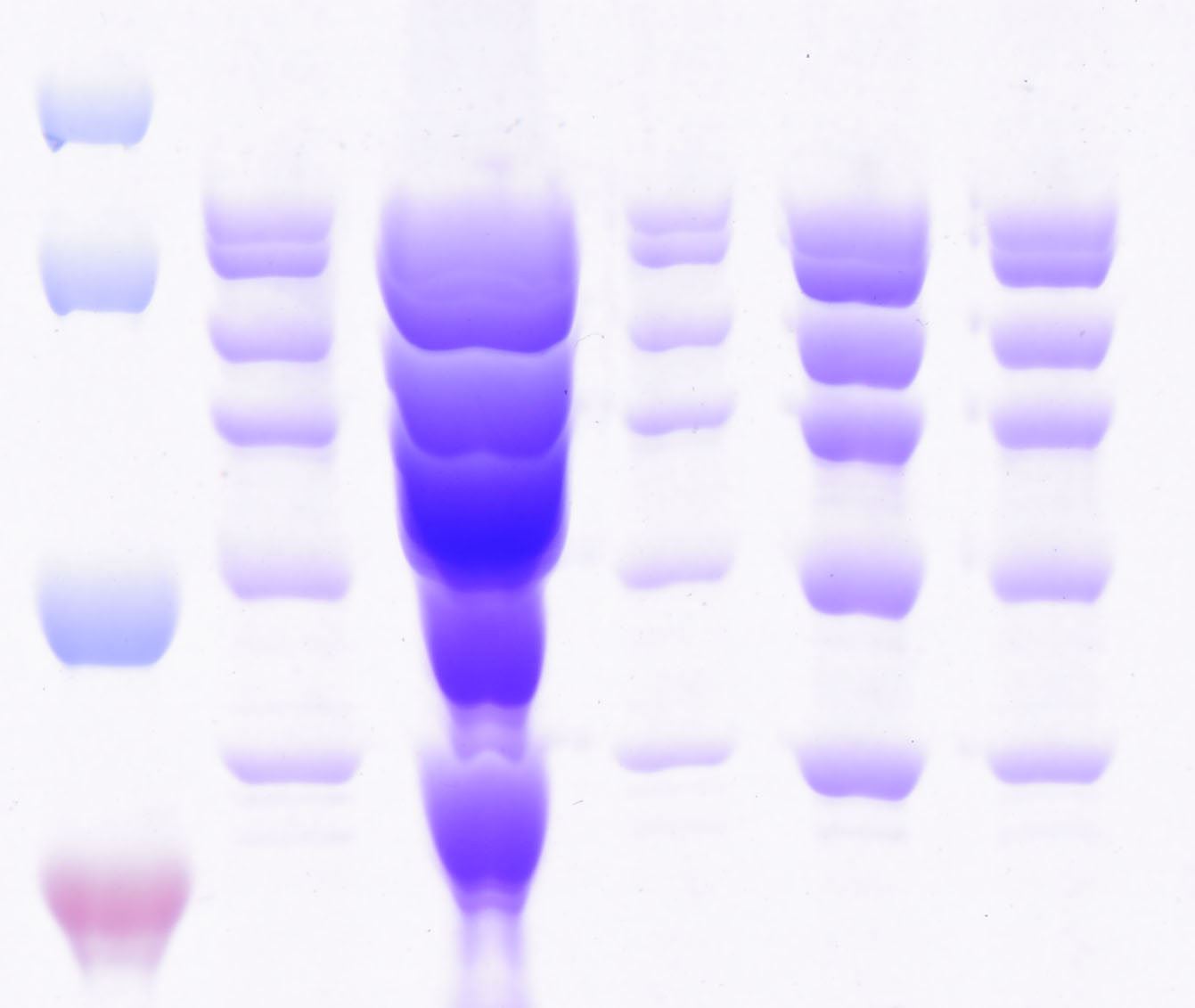

Supplement: Figure 1—source data 1. [file elife-80901-fig1-data1.zip › Figure 1-source data 1/Figure 1b and 1d/20211126_yeast_HOPS_Coomassie_gel_raw.jpg]

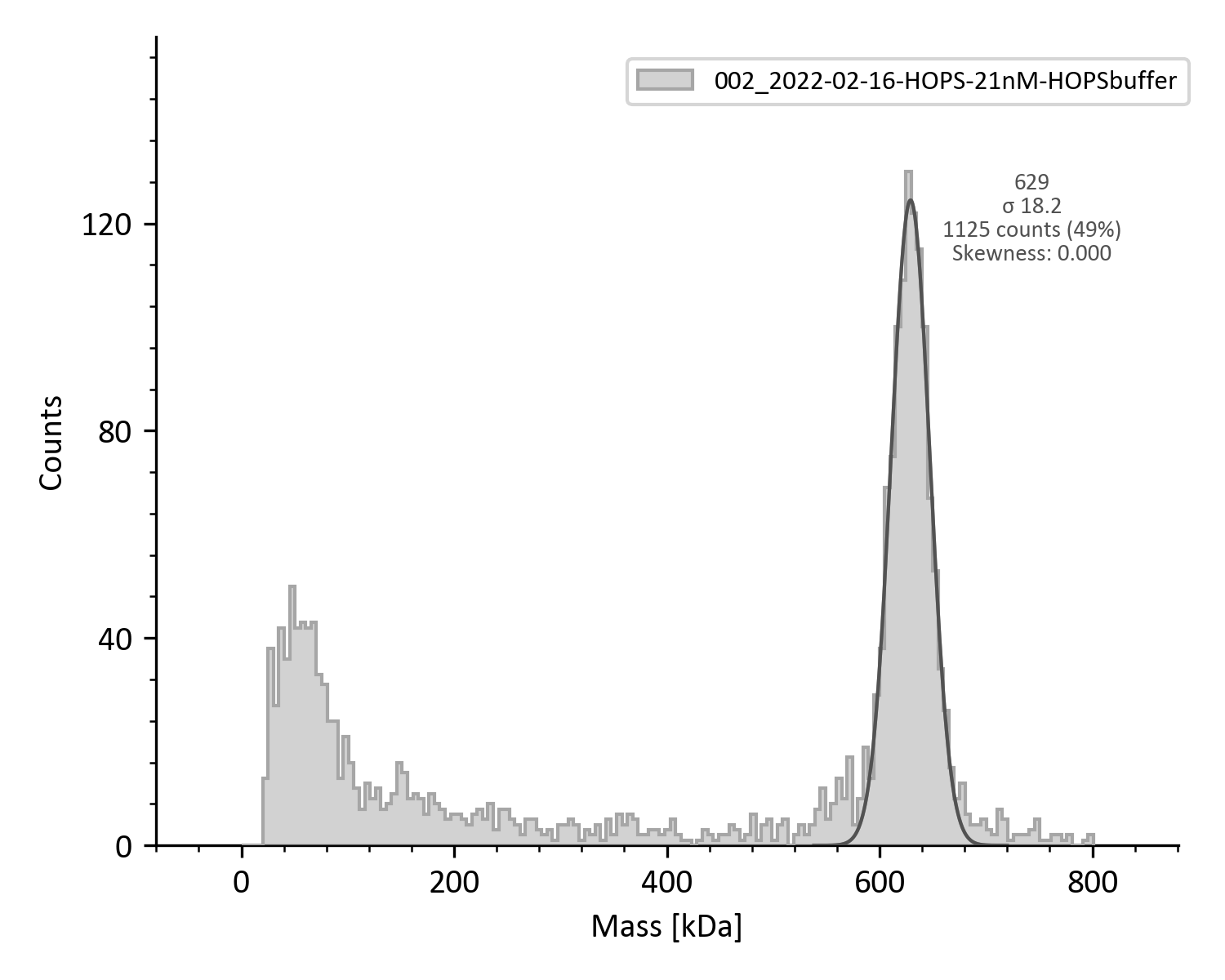

Supplement: Figure 1—source data 1. [file elife-80901-fig1-data1.zip › Figure 1-source data 1/Figure 1c/2022-02-16_HOPSwt_afterSEC_21nM.png]

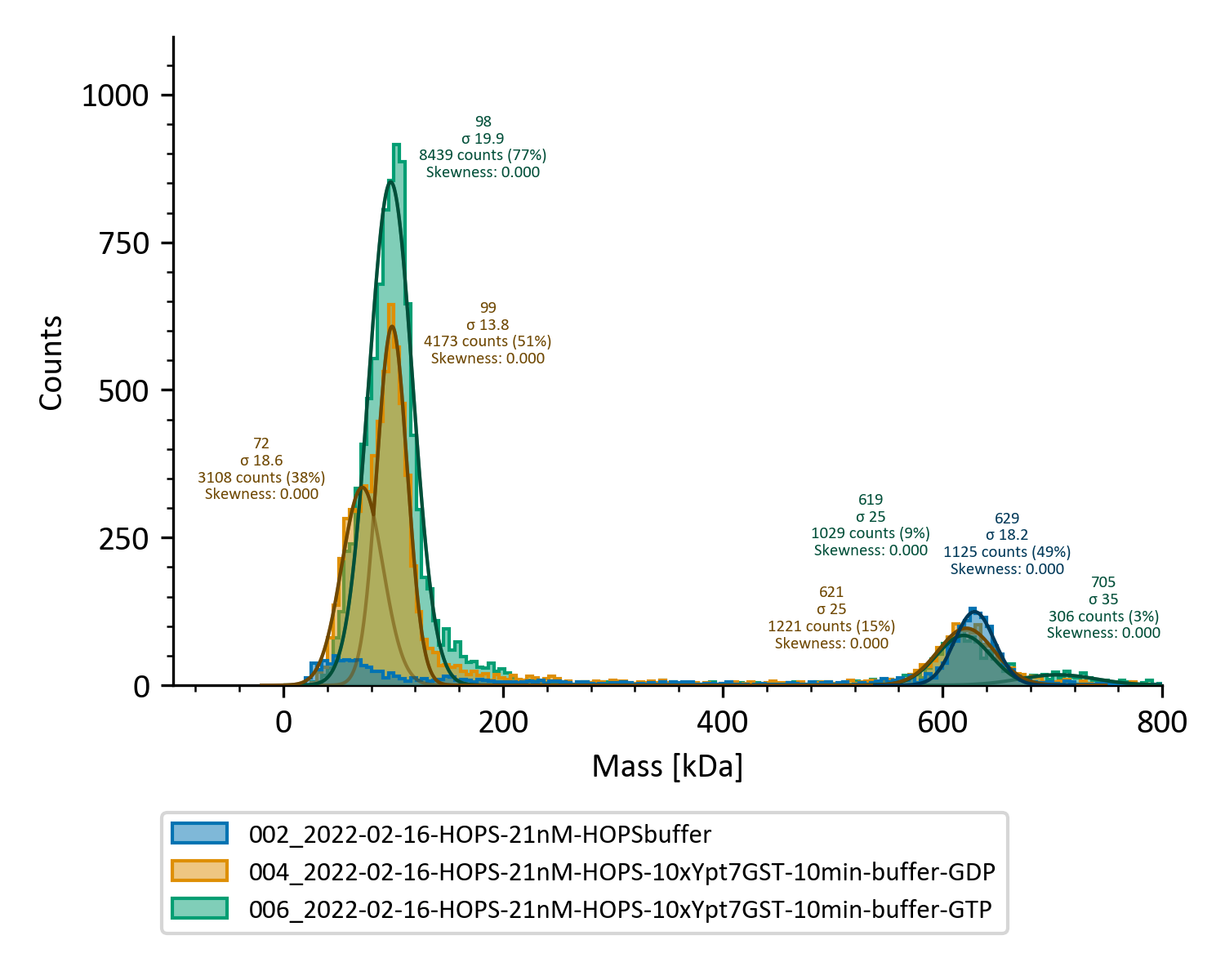

Supplement: Figure 1—source data 1. [file elife-80901-fig1-data1.zip › Figure 1-source data 1/Figure 1c/2022-02-16_HOPSwt_afterSEC_21nM_with-orwo-Ypt7-GST.png]

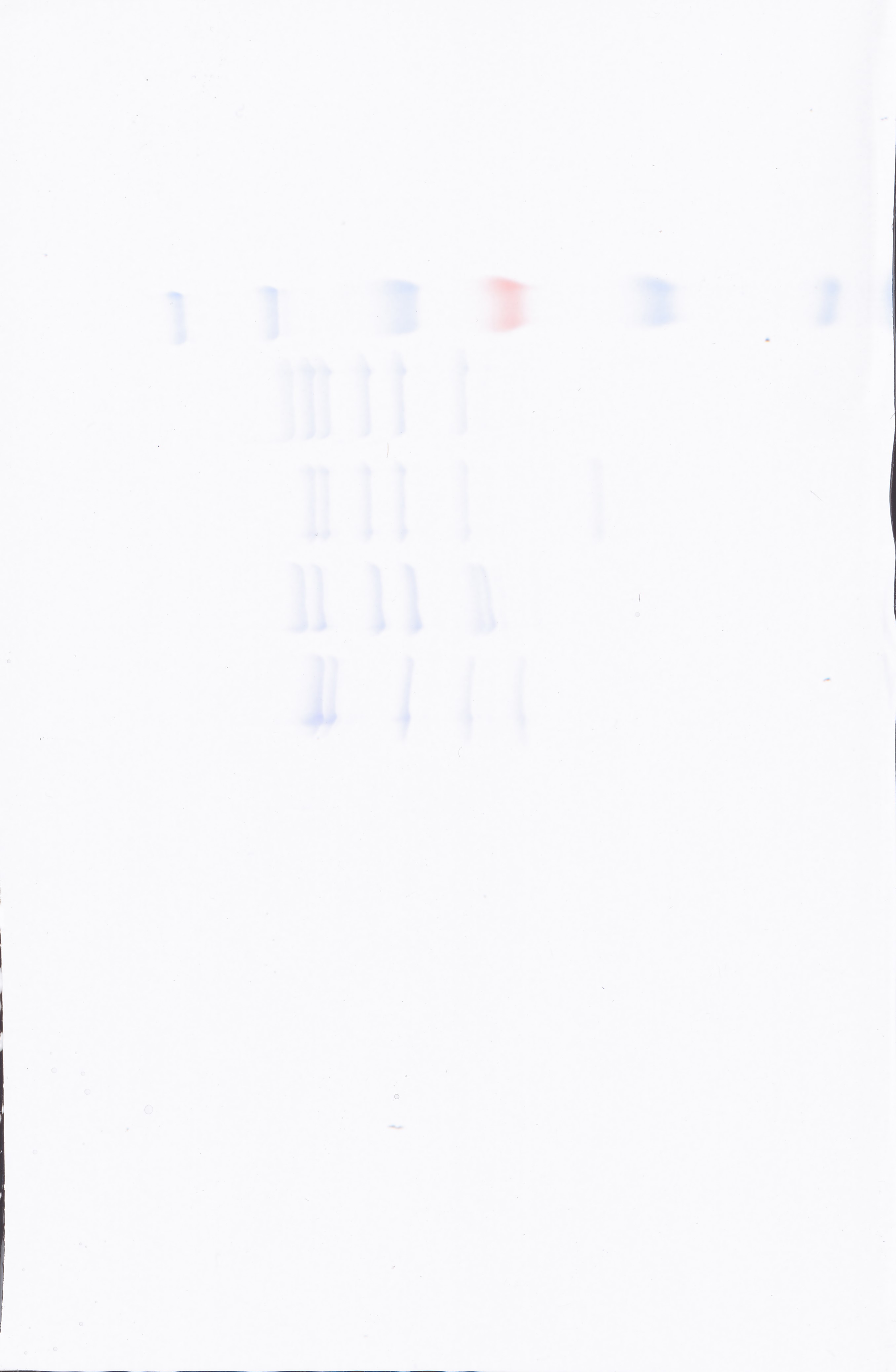

Supplement: Figure 1—figure supplement 4—source data 1. [file elife-80901-fig1-figsupp4-data1.zip › Figure 1-figure supplement 4-source data/Figure 1 S4a/HOPS mutants_raw.jpg]

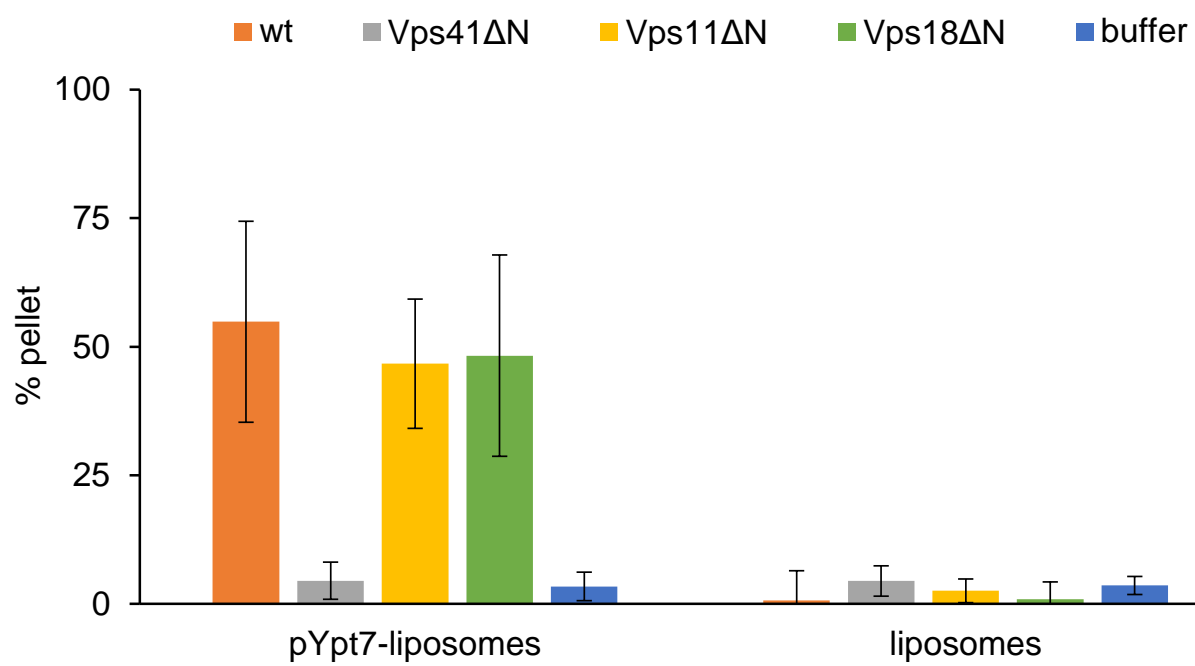

Supplement: Figure 3—source data 1. [file elife-80901-fig3-data1.zip › Figure 3-source data/Figure 3b/2022-04-11 Tethering assay_pYpt7-lipos_HOPS mutants_combined data.pdf]
