# Supplementary material for: Structure of the HOPS tethering complex, a lysosomal membrane fusion machinery
Source: eLife. 2022 Sep 13;11:e80901. doi: 10.7554/eLife.80901 (PMC9592082; doi:10.7554/eLife.80901)
Supplement: Supplementary file 1. [file elife-80901-supp1.docx]

**Supplementary File 1. Yeast strains used in the study**

| **Strain** | **Genotype** | **Source** |
| --- | --- | --- |
| SEY6210 | MATalpha *leu2-3,112 ura3-52 his3-∆200 trp-∆901 lys2-801 suc2-∆9 GAL* | Reggiori Lab |
| CUY2489 | MATalpha *his3-∆200 leu2-∆0 lys2-∆0 met15-∆0 trp1-∆63 ura3-∆0 VPS11::HIS3-GAL1pr VPS16::NatNT2-GAL1pr VPS18::KanMX-GAL1pr* | Ostrowicz et al., 2010 |
| CUY13050 | MATa *his3-∆200 met15-∆0 trp1-∆63 ura3-∆0 VPS41::TRP1-GAL1pr VPS39::KanMX-GAL1pr VPS33::HIS3-Gal1pr VPS41::HphNT1-FLAG* | This study |
| CUY13080 | CUY2489xCUY13050 | This study |
| CUY13079 | MATa *his3-∆200 leu2-∆0 met15-∆0 trp1-∆63 ura3-∆0 VPS39::KanMX-Gal1pr VPS33::HIS3-GAL1pr VPS41∆aa1-499::NatNT2-GAL1pr VPS41∆N::3xFLAG-HphNT1* | This study |
| CUY13194 | MATalpha *his3-∆200 leu2-∆0 lys2-∆0 met15-∆0 trp1-∆63 ura3-∆0 VPS11::HIS3-GAL1pr VPS16::NatNT2-GAL1pr VPS18::KanMX-GAL1pr-3HA VPS41::∆aa1-499-TRP1* | This study |
| CUY13195 | CUY13079xCUY13194 | This study |
| CUY13751 | MATalpha *his3-∆200 leu2-∆0 lys2-∆0 met15-∆0 trp1-∆63 ura3-∆0 VPS11::HIS3-GAL1pr VPS18::TRP-GAL1pr VPS39::KanMX-GAL1pr VPS39::FLAG-HphNT1* | This study |
| CUY13764 | Matalpha *his3-∆200 leu2-∆0 lys2-∆0 met15-∆0 trp1-∆63 ura3-∆0 VPS11∆aa1-349::URA-GAL1pr VPS16::NatNT2-GAL1pr VPS18::KanMX-GAL1pr-3HA VPS39::TRP1-GAL1pr VPS39::KanMX-GAL1pr VPS33::HIS3-Gal1pr VPS41::HphNT1-FLAG* | This study |
| CUY1908 | MATa *his3-∆200 met15-∆0 trp1-∆63 ura3-∆0 VPS392::TRP1-GAL1pr VPS41::KanMX-GAL1pr Vps33::HIS3-Gal1pr* | Ungermann lab |
| CUY13751 | MATalpha *his3-∆200 leu2-∆0 lys2-∆0 met15-∆0 trp1-∆63 ura3-∆0 VPS11::HIS3-GAL1pr VPS16::kanMX-GALpr1 VPS18∆aa1-335::TRP-GAL1pr VPS18::FLAG-HphNT1* | This study |
| CUY13763 | CUY1908xCUY13763 | This study |
| CUY2742 | Mat alpha *vps11-1(Ts)* *leu2-3,112 ura3-52 his3-∆200 trp1-∆901 lys2-801 suc2-∆9 VPS11-1::HIS3-HA* | Robinson et al. 1991 |
| CUY2743 | Mat alpha *vps11-3(Ts) leu2-3,112 ura3-52 his3-∆200 trp1-∆901 lys2-801 suc2-∆9* | Robinson et al. 1991 |
| CUY2744 | Mat alpha *vps18-1(Ts) leu2-3,112 his3-∆200 trp1-∆901 lys2-801 suc2-∆9* | Robinson et al. 1991 |
| CUY13557 | MATalpha *leu2-3,112 ura3-52 his3-∆200 trp-∆901 lys2-801 suc2-∆9 VPS41::ALFA-HphNT1* | This study |
| CUY13598 | Mat alpha *leu2-3,112 ura3-52 his3-∆200 trp1-∆901 lys2-801 suc2-∆9 VPS11-1::HIS3-HA VPS41::ALFA-HphNT1* | This study |
| CUY13559 | Mat alpha *leu2-3,112 ura3-52 his3-∆200 trp1-∆901 lys2-801 suc2-∆9 VPS41::ALFA-HphNT1* | This study |
| CUY13561 | Mat alpha *leu2-3,112 his3-∆200 trp1-∆901 lys2-801 suc2-∆9 VPS41::ALFA-HphNT1* | This study |

References

1. Ostrowicz, C. W. *et al.* Defined Subunit Arrangement and Rab Interactions Are Required for Functionality of the HOPS Tethering Complex. *Traffic* 11, 1334–1346 (2010).
2. Robinson, J.S. et al. A putative zinc finger protein, Saccharomyces cerevisiae Vps18p, affects late Golgi functions required for vacuolar protein sorting and efficient alpha-factor prohormone maturation. *Mol Cell Biol* 11(12), 5813-5824 (1991).
