# Supplementary material for: Structure of the HOPS tethering complex, a lysosomal membrane fusion machinery
Source: eLife. 2022 Sep 13;11:e80901. doi: 10.7554/eLife.80901 (PMC9592082; doi:10.7554/eLife.80901)
Supplement: Supplementary file 2. [file elife-80901-supp2.docx]

**Supplementary File 2. Cryo-EM data collection, refinement and validation statistics**

|  | HOPS upper  (EMDB-xxxx: consensus upper,  EMDB-xxxx: SNARE-binding,  EMDB-xxxx: backbone)  (PDB xxxx) | HOPS lower  (EMDB-xxxx: consensus lower,  EMDB-xxxx: bottom-Vps18,  EMDB-xxxx: bottom-Vps39)  (PDB xxxx) |
| --- | --- | --- |
| **Data collection and processing** |  |  |
| Magnification | 130,000 | 130,000 |
| Voltage (kV) | 200 | 200 |
| Electron exposure (e–/Å^2^) | 50 | 50 |
| Defocus range (μm) | -0.8 to -2.8 | -0.8 to -2.8 |
| Pixel size (Å) | 0.924 | 0.924 |
| Symmetry imposed | C1 | C1 |
| Initial particle images (no.) | 2,565,533 (after duplicates removal) | 2,565,533 (after duplicates removal) |
| Final particle images (no.) | 129,388 (129,389 for consensus upper) | 115,273 (115,272 for bottom-Vps39) |
| Map resolution (Å)  FSC threshold | 4.2 (consensus upper), 3.6 (SNARE-binding), 4.0 (backbone)  0.143 | 4.4 (consensus lower), 4.4 (bottom-Vps18), 5.0 (bottom-Vps39)  0.143 |
|  |  |  |
| **Refinement** |  |  |
| Initial model used | AlphaFold | AlphaFold |
| Model resolution range (Å)  FSC threshold | 3.6-5.0  0.143 | 3.6-5.0  0.143 |
| Map sharpening *B* factor (Å^2^) | -90.6 (consensus upper)  -76.3 (SNARE-binding)  -78.7 (backbone) | -111.4 (consensus lower)  -116.1 (bottom-Vps18)  -198.2 (bottom-Vps39) |
| Model composition  Non-hydrogen atoms  Protein residues | 25801  3581 | 25801  3581 |
| R.m.s. deviations  Bond lengths (Å)  Bond angles (°) | 0.005  0.979 | 0.005  0.979 |
| Validation  MolProbity score  Clashscore  Poor rotamers (%) | 2.08  11.78  0 | 2.08  11.78  0 |
| Ramachandran plot  Favored (%)  Outliers (%) | 91.87  0 | 91.87  0 |
|  |  |  |
