# Supplementary material for: Structure of the HOPS tethering complex, a lysosomal membrane fusion machinery
Source: eLife. 2022 Sep 13;11:e80901. doi: 10.7554/eLife.80901 (PMC9592082; doi:10.7554/eLife.80901)
Supplement: Supplementary file 3. [file elife-80901-supp3.docx]

**Supplementary File 3.** **Crystallographic data collection and refinement statistics (molecular replacement)**

|  | ***Ct*Vps39_1-500_ (SAD)**  (pdb code: XXX) |
| --- | --- |
| **Data collection** |  |
| X-ray source | EMBL, Hamburg, Germany, P13 |
| Wavelength (Å)  Space group  Cell dimensions | 0.97937  P21212 |
| *a*, *b*, *c* (Å) | 64.70, 102.75, 59.56 |
| Resolution (Å) | 43.82‑2.89 (3.07‑2.89)* |
| Total reflections  Multiplicity  Unique reflections  Completeness (%)  *R*_meas_ (%)  CC(1/2)  *I* / σ*I* | 114239 (16970)  6.72 (6.48)  17010 (2619)  99.1 (94.8)  23.1 (124.3)  99.3 (76.6)  7.70 (1.80) |
| Mosaicity (°)  Wilson *B*-factor (Å^2^) | 0.523  58.48 |
| **Refinement** |  |
| Resolution (Å) | 40.23‑2.89 |
| Reflections (working, test set) | 9264, 463 |
| *R*_factor_, *R*_free_ | 0.269, 0.298 |
| Completeness for range (%)  No. atoms | 99.1 |
| Protein  Solvent  Protein residues | 2681  10  345 |
| R.m.s. deviations |  |
| Bond lengths (Å) | 0.003 |
| Bond angles (°)  mean *B* value (Å^2^) | 0.551  58.1 |

*Values in parentheses are for highest-resolution shell.
